# The complete chloroplast genome of *Pouteria caimito*

**DOI:** 10.1080/23802359.2019.1660249

**Published:** 2019-09-02

**Authors:** Dejun Yang, Qiong Qiu, Linhong Xu, Yumei Xu, Yi Wang

**Affiliations:** a Institute of Tropical Forestry, Yunnan Academy of Forestry, Puwen Yunnan, People's Republic of China;; b Laboratory of Forest Plant Cultivation and Utilization, Yunnan Academy of Forestry, Kunming Yunnan, People's Republic of China

**Keywords:** *Pouteria caimito*, chloroplast, Illumina sequencing, phylogenetic analysis

## Abstract

The first complete chloroplast genome sequences of *Pouteria caimito* were reported in this study. The cpDNA of *P. caimito* is 158,937 bp in length, contains a large single-copy region (LSC) of 88,100 bp and a small single-copy region (SSC) of 18,631 bp, which were separated by a pair of inverted repeat (IR) regions of 26,103 bp. The genome contains 130 genes, including 85 protein-coding genes, 8 ribosomal RNA genes, and 37 transfer RNA genes. The overall GC content of the whole genome is 36.8%. Phylogenetic analysis of 15 chloroplast genomes within the order Ericales suggests that *P. caimito* is closely related to *Pouteria campechiana*.

*Pouteria caimito* belongs to the genus Pouteria in family Sapotaceae (Ericales) is distributed in the Amazon region and Latin American (Fernandes et al. 2013). *Pouteria caimito* is a tropical fruit tree and has high commercial value. Its fruit has either an ovoid or spherical shape, with pulp usually translucent and peel yellowish (Almeida et al. 2008). *Pouteria caimito* has also been used as a traditional medicine in Latin American, and the extracts from its leaves showed strong radical-scavenging activity (França et al. 2016). It has been widely cultivated in Hainan and Jinghong, Yunnan Province of China since 2009, and planting has rapidly expanded (Duan et al. 2018). However, there have been no genomic studies on *P. caimito*.

Herein, we reported and characterized the complete *P. caimito* plastid genome (MN065160). One *P. caimito* individual (specimen number: 201804011) was collected from Jinghong, Yunnan Province of China (22°49′48ʺ N, 101°9′37ʺ E). The specimen is stored at Yunnan Academy of Forestry Herbarium, Kunming, China and the accession number is YAFH0012872. DNA was extracted from its fresh leaves using DNA Plantzol Reagent (Invitrogen, Carlsbad, CA, USA).

Paired-end reads were sequenced by using Illumina HiSeq system (Illumina, San Diego, CA). In total, about 29.1 million high-quality clean reads were generated with adaptors trimmed. Aligning, assembly, and annotation were conducted by CLC de novo assembler (CLC Bio, Aarhus, Denmark), BLAST, GeSeq (Tillich et al. 2017), and GENEIOUS v 11.0.5 (Biomatters Ltd, Auckland, New Zealand). To confirm the phylogenetic position of *P. caimito*, other 14 species of Order Ericales from NCBI were aligned using MAFFT v.7 (Katoh and Standley 2013) and maximum likelihood (ML) bootstrap analysis was conducted using RAxML (Stamatakis 2006); bootstrap probability values were calculated from 1000 replicates. *Alangium chinense* (MG524996) and *Cornus controversa* (KU852492) were served as the out-group.

The complete *P. caimito* plastid genome is a circular DNA molecule with the length of 158,937 bp, with a large single copy (LSC: 88,100 bp), small single copy (SSC: 18,631 bp), and two inverted repeats (IRa and IRb: 26,103 bp each). The overall GC content of the whole genome is 36.8%, and the corresponding values of the LSC, SSC, and IR regions are 34.6, 30.3, and 42.9%, respectively. The genome contains 130 genes, including 85 protein-coding genes, 8 ribosomal RNA genes, and 37 transfer RNA genes. Phylogenetic analysis showed that *P. caimito* clustered together with *Pouteria campechiana*, which indicated the phylogenesis classification of *P. caimito* (Figure 1). The determination of the complete plastid genome sequences provided new molecular data to illuminate the Ericales evolution.

**Figure 1.**
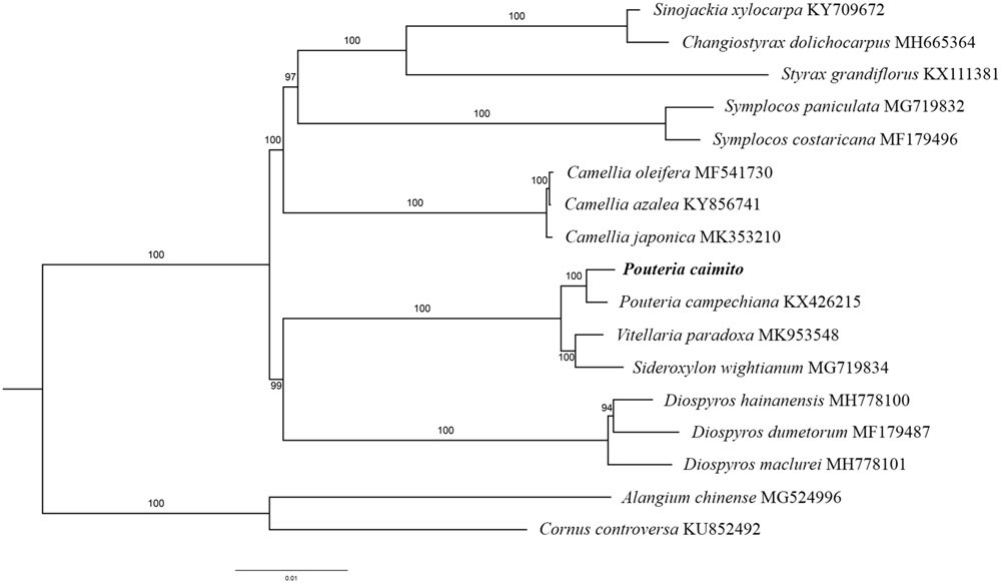
The maximum-likelihood tree based on the 15 chloroplast genomes of order Ericales. The bootstrap value based on 1000 replicates is shown on each node.
